# TRA-1-60-positive/CD45^low^ cells found in the peripheral blood of prostate cancer patients with metastatic disease – A proof-of-concept study

**DOI:** 10.1016/j.heliyon.2020.e03263

**Published:** 2020-01-24

**Authors:** Claudia Schäfer, Yawen Ju, Youngbin Tak, Cesar Vazquez, Sangyoon J. Han, Edwin Tan, Jerry W. Shay, Mats Holmqvist, Gaudenz Danuser, William M. Schopperle, Glenn Bubley

**Affiliations:** a Department of Cell Biology, University of Texas, Southwestern Medical Center, Dallas, TX, USA; b CureMeta, Boston, MA, USA; c Genitourinary Oncology, Beth Israel Deaconess Medical Center, Boston, MA, USA; d College of Engineering, Michigan Tech University, Houghton, MI, USA

**Keywords:** Oncology, Cancer research, Cancer, Diagnostics, TRA-1-60, Metastasis, Embryonic stem cells

## Abstract

**Purpose:**

Over 90% of all cancer related deaths are due to metastasis. However, current diagnostic tools can't reliably discriminate between invasive and localized cancers.

**Patients and methods:**

In this proof-of-concept study, we employed the embryonic stem cell marker TRA-1-60 (TRA+) to identify TRA + cells within the blood of prostate cancer patients and searched for TRA + cells in men with metastatic and localized cancers. We isolated whole peripheral blood mononuclear cells from 26 metastatic prostate cancer patients, from 13 patients with localized prostate cancer and from 17 healthy controls. Cells were stained for DAPI, CD45 and TRA + by immunofluorescence and imaged by epi-fluorescence microscopy. Imaged-based software was used both to identify TRA + cells, and to analyze CD45 levels in TRA+ and negative cells.

**Results:**

We found high numbers of TRA + cells within the blood of metastatic cancer patients, whereas healthy individuals or men with localized prostate cancer showed none or very low numbers of TRA + cells. Further analysis of the CD45 levels of TRA + cells revealed a small population of TRA + cells with almost undetectable CD45 levels that were found frequently in metastatic prostate cancer patients. By excluding CD45 positive cells from the TRA + cell pool, we were able to refine the assay to be highly specific in identifying men with metastatic disease. In fact, the difference of CD45 levels between TRA+ and negative cells was a robust measure to distinguish between men with localized and metastatic prostate cancers in this small patient cohort.

**Conclusions:**

The data suggest that metastatic prostate cancer patient have significant numbers of TRA+/CD45^low^ cells which might represent a potential tool for diagnostic assessment in the future.

## 1 Introduction

Metastatic spread from the primary tumor to a distant organ site is the leading cause of cancer-related deaths. Prostate cancer is the third deadliest type of cancer in men, behind lung and colorectal cancer (source: cancer.org). The standard treatment for men with localized disease had been prostatectomy and/or different forms of radiation therapy as well as testosterone deprivation [1]. More recently active surveillance strategies have been pursued in men to counteract over treatment. Despite primary treatment with either or both modalities a substantial number of men will develop metastatic disease. Metastatic prostate cancer will inevitably progress to castration-resistant prostate cancer [2, 3]. In prostate cancer, as in other solid tumors, the ability to predict disease progression with a simple blood test would be of great utility, both for diagnostic reasons and as a powerful surveillance tool. Such an assay would provide clinicians with a tool to identify patients with malignant disease progression at an earlier time point when treatment efforts are more effective.

Podocalyxin is a member of the CD34 family [28]. In healthy tissues, podocalyxin functions as a regulator for cell-cell contacts and can be found in podocytes of the kidney, within epithelial and vascular cells as well as on haematopoietic progenitor stem cells [29, 30, 31, 32]. During cancer progression, podocalyxin plays an important role in extravasation and intravasation of cancer cells [33]. Expression of podocalyxin in various cancers such as prostate, breast and gastric highly correlates with malignant progression and metastasis [34, 35]. TRA-1-60 is a carbohydrate addition to podocalyxin and is highly expressed on normal pluripotent stem cells [10]. For decades TRA-1-60 has been the gold standard surface marker for human embryonic stem cells [9].

Recent efforts to provide better patient's outcome predictions incorporated embryonic stem cell signatures [4]. Such embryonic or pluripotent stem cell profiles were found in many cancers such as breast cancer, glioblastoma, bladder carcinomas [5], in malignant cervical cancer [6], in brain, lung and colon cancer [7] as well as in prostate cancer [8]. All these signatures correlated with poor prognosis. There are increasing evidences in the literature that TRA-1-60 correlates with metastatic progression in prostate cancer and many other cancers [11, 12, 13, 14, 15, 16]. Based on this, we tested the potential of TRA-1-60 expression as a marker to develop a blood test which is able to discriminate aggressive, metastatic prostate cancer from localized prostate cancer and further to predict disease progression. In summary this study demonstrates the potential of TRA-1-60 to be a powerful biomarker in order to identify men with metastatic disease, in contrast to men with localized prostate cancer.

## 2 Material and methods

### 2.1 Patient samples and PBMCs isolation

Patient samples were derived from a tissue bank study which in consented patients agreed to data collection and serum and whole-blood samples (BIDMC protocol 2001P-002244, Prostate Cancer Tissue Bank and Database). PBMCs were isolated from whole blood by Ficoll gradient. Blood was diluted 1:1 with sterile PBS and added on top of Ficoll-Paque PLUS (GE), followed by spinning at 1600rpm for 30 min at 4 °C. PBMCs were collected, washed with PBS and fixed with 4% PFA for 10 min at room temperature (RT).

### 2.2 Staining methods

Cells were fixed in suspension using 4% PFA at RT for 10min and blocked with 10% goat serum, 3% BSA and Fc blocker (BD Pharmingen, 1:10) in PBS for 30 min at RT. PBMC were incubated with CD45-FITC (eBioscience, 1:20) and TRA-1-60 (Life technologies, 1:50) in blocking at 4 °C overnight and then stained with Alexa Fluor®594 Anti-Mouse (Thermo Fisher, 1:200) for 1 h at RT. The negative control was only incubated with Alexa Fluor®594 Anti-Mouse. Cells were stained with DAPI and mounted onto slides.

### 2.3 Imaging

Imaging was performed on a fluorescence Eclipse microscope (Nikon) equipped with a sCMOS camera, SOLA system and a motorized filter turret for DAPI, FITC and TexasRed using a 10x Plan Fluor objectives. For each patient maximal 20 areas and 10 areas of the control were imaged. The field of view for each area was 1664 μm × 1404 μm with a pixel size of 0.65μm. Exposure times were kept constant between the stained slides and the controls.

### 2.4 Image analysis

DAPI images were pre-processed with Gaussian smoothing (sigma = 1 pixel) and background subtraction. Otsu thresholding was used to generate a mask. To separate segmentations containing more than one nucleus, the watershed algorithm was applied. This mask was used as a seed label. For efficient processing, segmentations whose areas are larger than the median of all segmentations were considered for watershed.

To capture the TRA-1-60 signal, areas per each identified nucleus were enlarged. For this, a new mask was created by Rosin-thresholding of the pre-processed image yielding a threshold value for DAPI images smaller than a value from Otsu's method. The mask was dilated by five pixels to ensure coverage of the entire cell. To label individual nuclei, the labels obtained in the seed label were propagated to the dilated mask that each pixel is attached with the closest label.

The threshold was defined for each patient using the negative control and setting the detection levels to zero. This threshold was applied to the images stained for TRA-1-60. The segmented mask was compared with the dilated nuclei mask to count the number of nuclei that contain pixels from the TRA-1-60 signal. A minimal signal threshold of at least 5 pixels was applied to all images. CD45 intensity levels were analyzed inside each dilated nuclei mask for TRA-1-60 positive and negative cells separately.

We extracted 3 different measurements: 1) percentage of TRA + cells per image. 2) The number of cells that are both TRA+ and CD45^low^ was extracted. Mean CD45 levels from all TRA negative cells were calculated for each patient, which was used to normalize the CD45 levels for all TRA + cells. Normalized CD45 levels below 0.3 were considered CD45^low^. These numbers were then expressed as numbers of cells normalized to per 1000 cells. 3) The differences of CD45 levels from TRA + cells subtracted by the CD45 levels of TRA negative cells. Only patients with at least three TRA + cells were included into this analysis.

To calculate the background fluorescence intensity of the CD45 staining we used the negative control and quantified the mean fluorescence intensities in the dilated nuclei mask areas. This was calculated exemplarily in 6 patients and shown in Figure 5.

### 2.5 Data presentation

Data were analyzed and bar graphs were made using Excel (Microsoft). ROC curve analysis was performed using the Prism6 software (GraphPad).

## 3 Results

**Figure 1:**
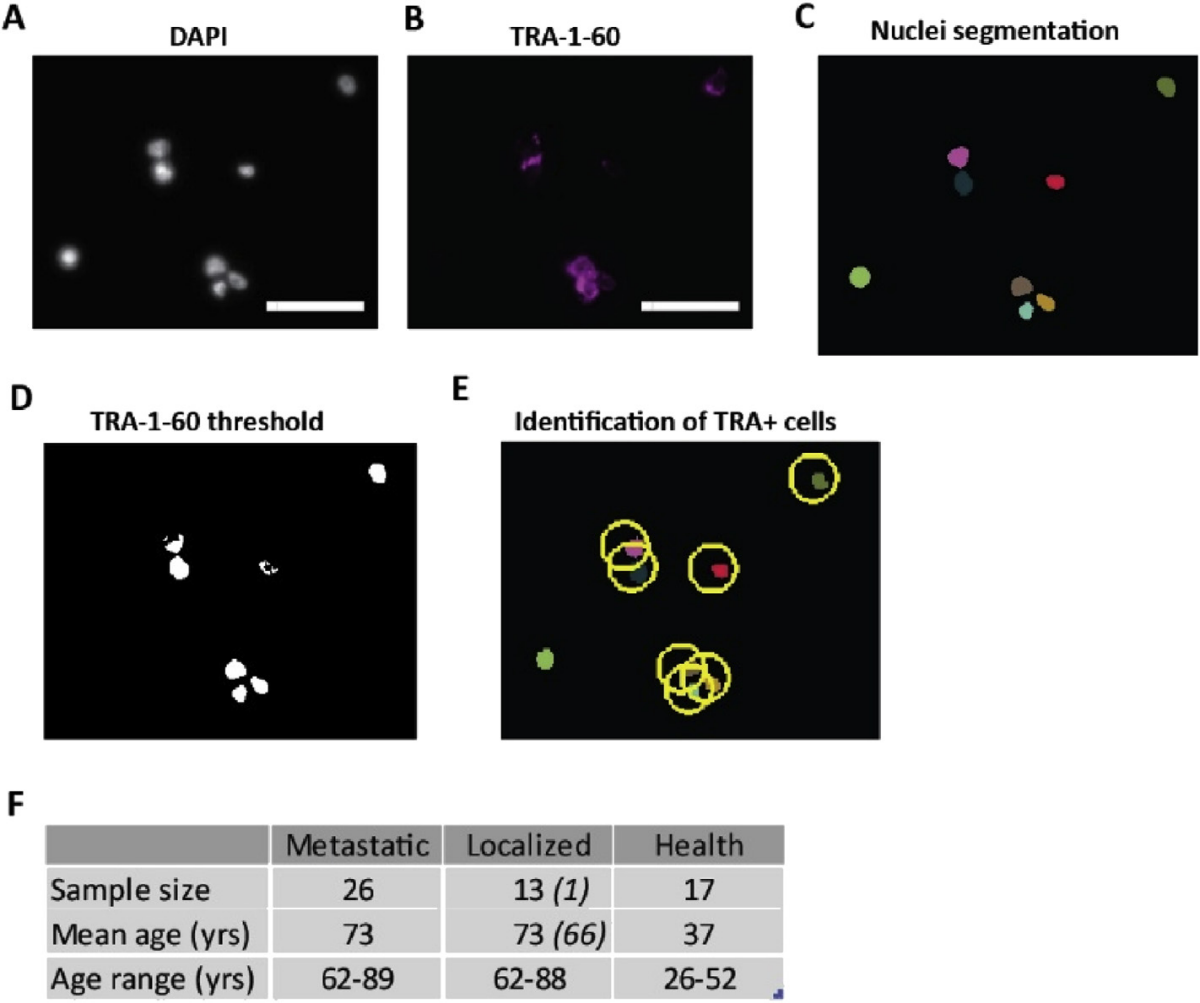
Validation of the software and overview of sample cohort– The embryonal carcinoma cell line Tera-1 was used as positive control to validate the software (A-E). Cells were fixed and stained for DAPI (A) and TRA-1-60 (B), Scale bar = 50 μm. These images were analyzed using the custom-made software. First, the DAPI image was used to segment the nuclei (C). Next, the TRA-1-60 signal was thresholded (D) and overlaid with the nuclei segmentation to identify TRA-1-60 + cells (E, TRA + cells circled in yellow). (F) Overview of the sample cohort. The italic numbers refer to a patient that was categorized localized but progressed during the study to metastatic. yrs = years.

The goal of this study was to test if the embryonic marker TRA-1-60 could be used to discriminate prostate cancer patients with localized or metastatic diseases. For this purpose, blood samples from patients with localized or metastatic prostate cancer and from healthy controls were collected at *Beth Israel Deaconess Medical Center* (*BI*), de-identified and blinded. As a first attempt we tested the FDA-cleared CELLSEARCH® platform for our purpose in collaboration with the diagnostics team at Jansson. The CELLSEARCH® CXC kit was used to detect circulating tumor cells (CTCs) based on the expression of epithelial cell adhesion molecule (EpCAM). CTCs were then stained for DAPI, Cytokeratin (CK), CD45, a marker for white blood cells, and TRA-1-60. Based on the classification of being EpCAM+/CK+/CD45-we detected ≥5 CTCs in two out of 10 analyzed blood samples from patients with metastatic prostate cancer. Adding TRA-1-60 expression (TRA+) to the analysis (EpCAM+/CK+/CD45-/TRA+) we were only able to identify one patient with metastatic prostate cancer (out of 10) by detection of ≥5 CTCs (data not shown). Since the isolation of CTCs from metastatic prostate cancer patients was not very effective and infrequent we decided to take a more unbiased approach without any CTC capturing step by using whole peripheral mononuclear cells (PBMCs) for our analysis. Additionally, we switched from a flow-based analysis to direct imaging of the stained PBMCs by fluorescence microscopy combined with high-throughput image analysis software. Whole PBMCs were isolated, stained for DAPI, CD45 and TRA-1-60 and then mounted on imaging slides. The slides were imaged by fluorescence microscopy and then analyzed by custom-made software. In summary, the analysis extracted the total number of cells analyzed; the percentage of TRA + cells and the mean CD45 intensities for each TRA+ and negative cells. To validate the software we used the embryonal carcinoma cell line Tera-1 since these cells are known to express TRA-1-60 [10]. Tera-1 cells were stained for DAPI (Figure 1A) and TRA-1-60 (Figure 1B), imaged and analyzed with our platform (Figure 1C–E). The nuclei segmentation is shown in Figure 1C and the TRA-1-60 signal after thresholding is shown in Figure 1D. Once overlaid the TRA + cells were identified (Figure 1E). In two independent experiments, the software recognized 95.5% or 91.8% of all analyzed Tera-1 cells as TRA + cells.

The study cohort comprised 26 patients diagnosed with metastatic prostate cancer with an age range from 62 to 89 years (Figure 1F). Most of these patients were undergoing active treatment during the course of sample collection. The study also included 13 patients diagnosed with localized, non-metastatic prostate cancer patients ranging from 62 to 88 years of age and one patient who progressed from localized to metastatic prostate cancer during the study (age 66). Additionally, we included 17 healthy control samples with an age range from 26 to 52 years.

**Figure 2:**
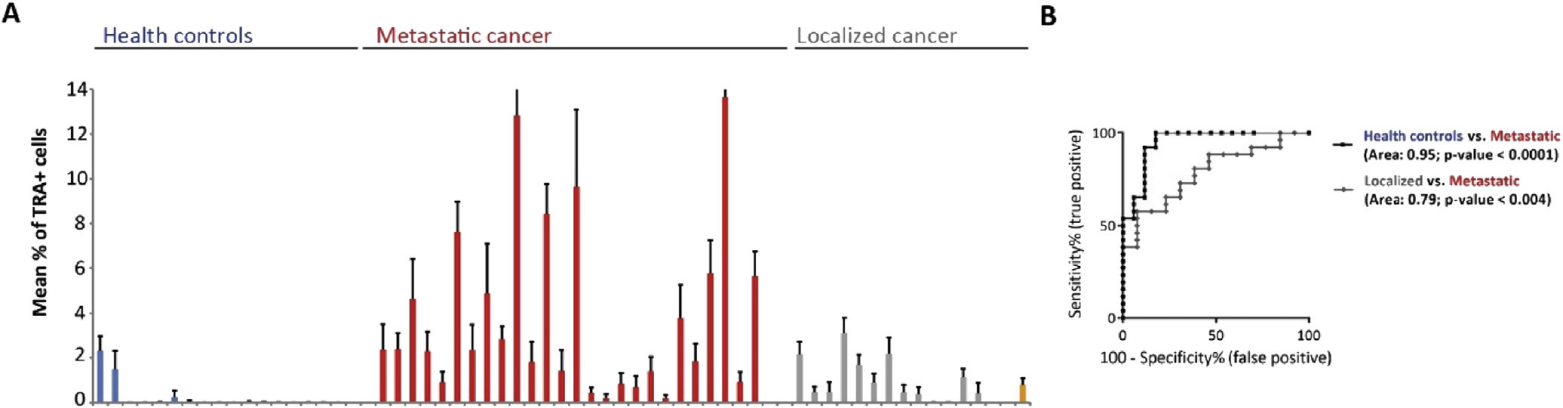
Identification of TRA-1-60+ cells in the blood of healthy controls, metastatic and localized prostate cancer patients – (A) The mean percentage of TRA-1-60 positive (TRA+) cells were analyzed from whole peripheral blood mononuclear cells taken from 17 healthy controls (blue), 26 metastatic (red), 13 localized (gray) prostate cancer patients and 1 (orange) localized prostate cancer patient who progressed to metastatic disease during the study. Error bars indicate standard deviation from n = 20. (B) Receiver operating characteristics (ROC) curves shows diagnostic value and area under the curve comparing the percentages of TRA + cells from healthy controls and metastatic prostate cancer patients (black curve) and the percentages from localized and metastatic prostate cancer patients (gray curve).

We initially compared the mean percentages of TRA + cells found in healthy controls with the mean percentages seen in metastatic or localized prostate cancer patients (Figure 2A). Healthy controls (Figure 2A, blue) showed mostly low numbers of TRA + cells ranging from 0% to 2.3%. In 14 of the 17 samples the mean percentage was below 0.04% with five of them showing no TRA + cell. In comparison, samples from metastatic prostate cancer patients contained higher numbers of TRA + cells ranging from 0.2% to up to 13.6% (Figure 2A, red). 24 out of the 26 samples showed numbers well above 0.24% with 17 samples having more than 1.5% of TRA + cells. None of the samples from metastatic prostate cancer patients was scored 0%. The samples from localized prostate cancer patients ranged from 0.002% to 3.1% of TRA + cells (Figure 2A, gray). Nine of the 13 samples were scored below 1.5% of TRA + cells. The patient who progressed from localized to metastatic prostate cancer during the study showed 0.8% of all analyzed cells being positive for TRA-1-60 (Figure 2A, orange).

Receiver operating characteristic (ROC) curve analysis was used to test the discriminating power of the percentage of TRA + cells as a diagnostic test (Figure 2B). This determined a significant diagnostic performance to distinguish between healthy controls and metastatic prostate cancer patients with the area under the curve (AUC) of 0.95, the confidence interval (CI) between 0.88-1 and a *p*-value < 0.0001 (Figure 2B, black curve). Comparing the mean percentages of TRA + cells from localized with metastatic prostate cancer patients showed an AUC of 0.79, a CI between 0.64-0.93 and a *p*-value < 0.004 (Figure 2B, gray curve). Even though this measure significantly discriminated between localized and metastatic prostate cancer it only shows a fair diagnostic performance.

**Figure 3:**
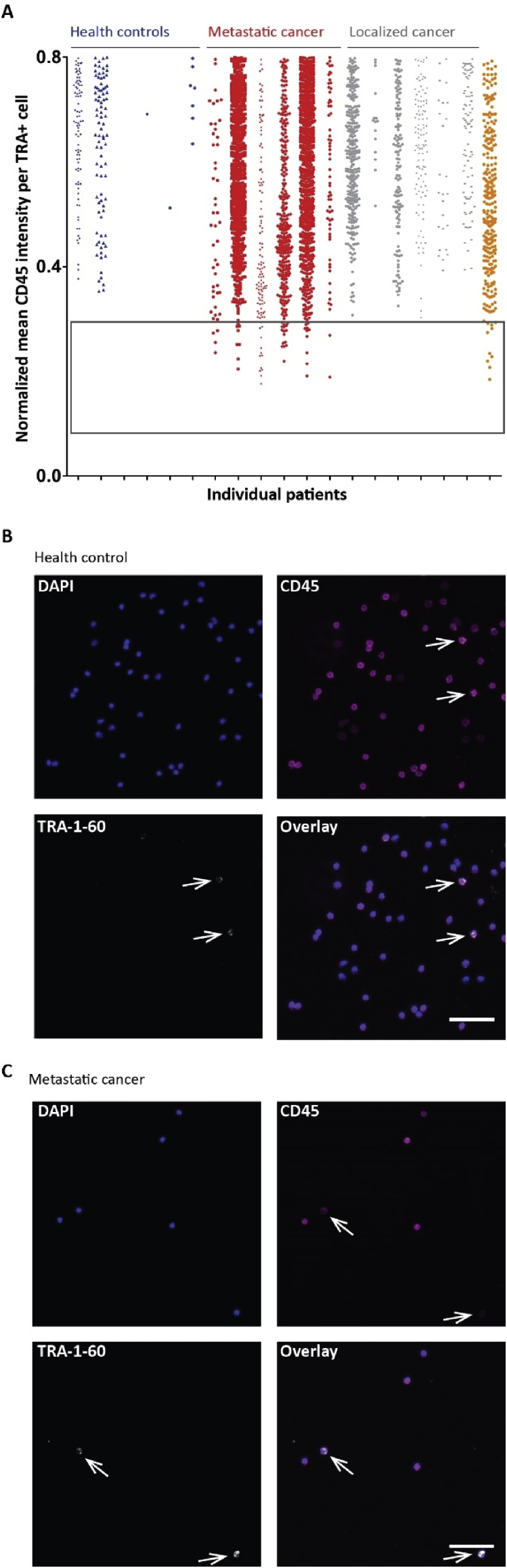
TRA-1-60 positive and CD45^low^ cell population is present in metastatic prostate cancer patients – (A) Boxplots show normalized mean CD45 intensities for TRA-1-60 positive (TRA+) cells detected per sample ranging from level 0 to 0.8. Each dot represents one TRA + cell. Data from six healthy controls (blue), six metastatic (red) and six localized (gray) prostate cancer patients are exemplified. The patient that progressed from localized to metastatic during the study is shown in orange. The box highlights the TRA+/CD45^low^ cell population. Staining for DAPI (blue), CD45 (purple) and TRA-1-60 (white) in cells from healthy control (B) and metastatic prostate cancer patient (C) show TRA + cell indicated by the white arrows. Scale bar = 50 μm. Note that TRA + cells in healthy controls show high levels of CD45 and low levels in patients with metastatic prostate cancer.

TRA + cells were detected in all cohorts, although the number of cells was higher in patient with metastatic disease. Next, we wanted to know if there is a difference between the cells from the cohorts. Therefore, we analyzed the mean CD45 levels of all TRA + cells and blotted the mean CD45 intensities for TRA + cells from six healthy controls (Figure 3A, blue), six metastatic (Figure 3A, red), six localized (Figure 3A, gray) prostate cancer patients and from the one patient who progressed (Figure 3A, orange) so that each dot represents one TRA + cell. We found a small population of TRA + cells that had very low CD45 levels (close to background levels) which was only present in metastatic prostate cancer patients (Figure 3A, box) and in the one patient who progressed to metastatic prostate cancer. To investigate this in more detail, we checked the original images and indeed, TRA + cells in healthy controls showed a strong CD45 signal (Figure 3B, white arrows) whereas in metastatic prostate cancer patients the TRA + cells stained remarkably less for CD45 (Figure 3C, white arrows).

**Figure 4:**
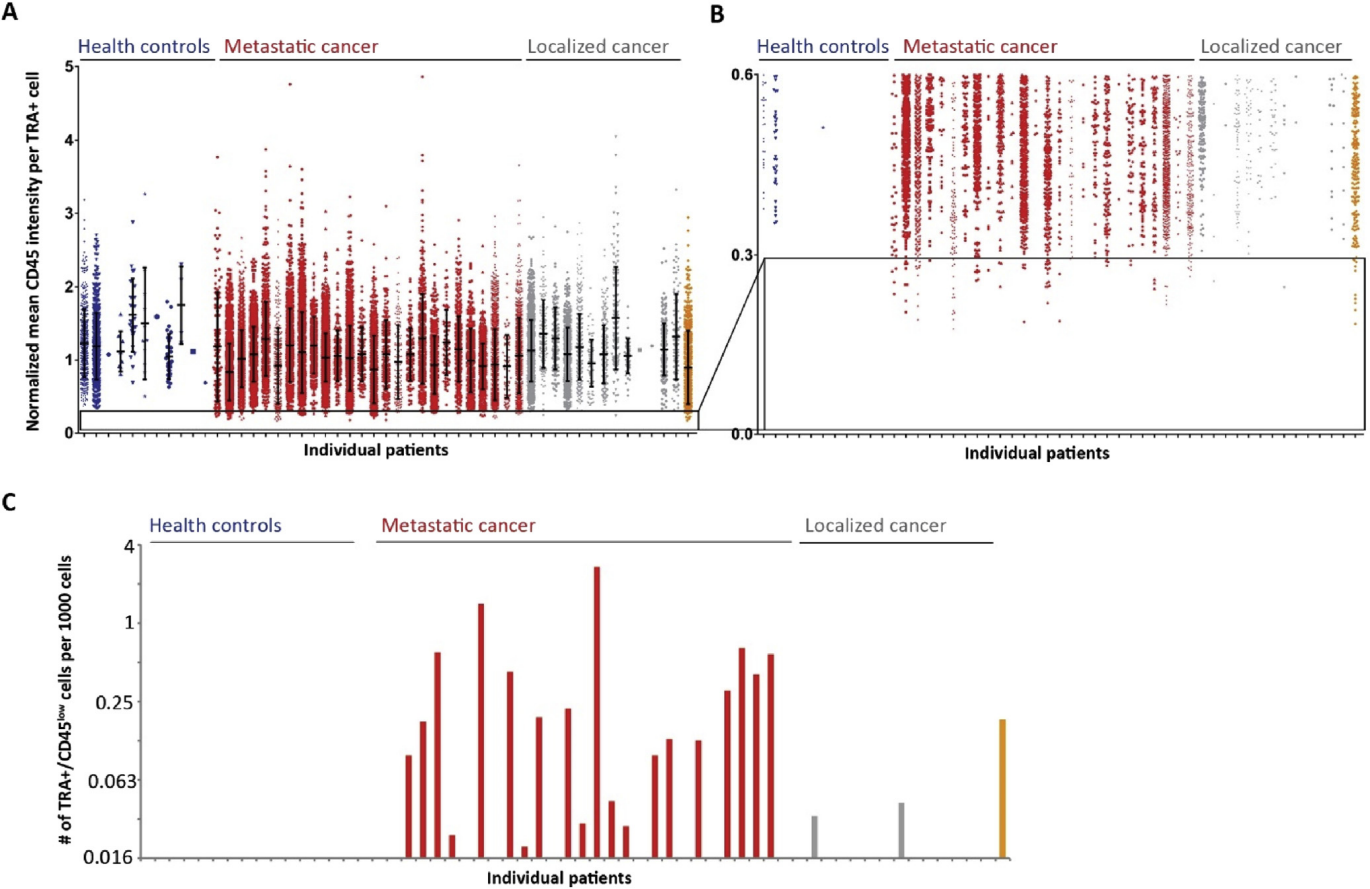
TRA-1-60+/CD45^low^ cell population highly correlates with metastatic prostate cancer – (A) Complete data set of normalized mean CD45 intensities for all TRA-1-60 positive (TRA+) cells detected per sample. Each dot of the boxplots represents one TRA + cell. The black lines indicate the mean and the whiskers the standard deviation. Healthy controls (blue), metastatic (red) and localized (gray) prostate cancer samples. The patient that progressed from localized to metastatic during the study is shown in orange. (B) shows a zoom in of the data shown in (A) highlighting the TRA+/CD45^low^ cell population. In (C) the counts of TRA+/CD45^low^ cells were normalized to the total number of cells analyzed and displayed as number of TRA+/CD45^low^ cells per 1000 cells for each healthy control (blue), metastatic (red), localized (gray) prostate cancer samples and for the patient who progressed (orange).

**Figure 5:**
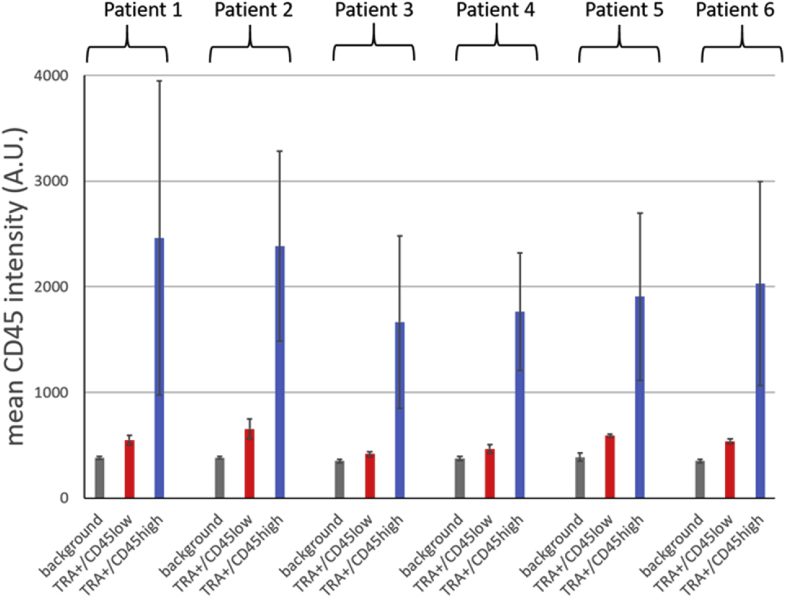
Mean CD45 fluorescence intensity was analyzed in 6 datasets from 6 different patients (1–6). Background intensity (grey) was calculated from PBMCs treated with only secondary antibody. CD45 levels were analyzed from PBMCs stained for CD45 and CD45 mean intensities were blotted for TRA-1-60+/CD45low (red) and TRA-1-60+/CD45high (blue) population. Standard errors show standard deviation.

Next, we plotted the normalized CD45 levels for all TRA + cells found in each of the samples (Figure 4A). As seen before (Figure 3A), TRA + cells showed a wide range of CD45 levels in all cohorts. We frequently found TRA + cells with a mean CD45 intensity below 0.3 AU in metastatic prostate cancer patients (Figure 4B, red) as well as in the patient who progressed from localized to metastatic prostate cancer during the study (Figure 4B, orange). This population of TRA + cells and almost undetectable CD45 levels (TRA+/CD45^low^) here further defined as below 0.3, was completely missing in all healthy controls (Figure 4B, blue). The CD45 levels are reported here as low and not as negative since these cells still showed a slightly higher mean fluorescence intensity compared to the background staining (Figure 5). Because of this minimal intensity we could not exclude residual CD45 expression on these cells. More importantly, only two cells meeting these criteria were found in two of 13 patients with localized prostate cancer (Figure 4B, gray). Since the total number of cells analyzed varied from sample to sample, we normalized the numbers of TRA+/CD45^low^ cells found in each sample to the total amount of cells analyzed and expressed this measure as TRA+/CD45^low^ cells per 1000 cells (Figure 4C). Only two patients with localized prostate cancer had 0.03 or 0.04 TRA+/CD45^low^ cells per 1000 cells (Figure 4C, gray). In comparison, we found TRA+/CD45^low^ cells in 20 out of 26 patients with metastatic prostate cancer (Figure 4C, red). In two patients we found high numbers of 1.4 and 2.7 TRA+/CD45^low^ cells per 1000 cells. The patient who progressed from localized to metastatic prostate cancer during the study had 0.18 of TRA+/CD45^low^ cells per 1000 cells (Figure 4C, orange).

**Figure 6:**
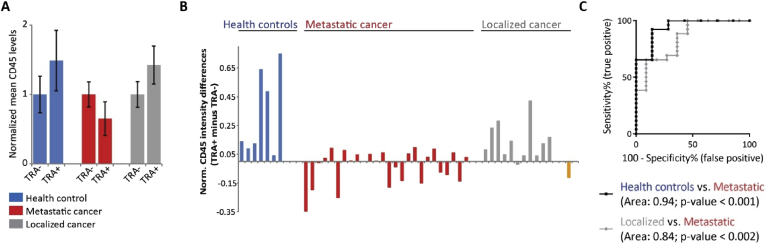
Difference in CD45 levels of TRA-1-60 positive minus negative cells is a good diagnostic measure to distinguish between localized and metastatic prostate cancer – (A) Mean CD45 levels in TRA-1-60 negative (TRA-) are lower compared to TRA-1-60 positive (TRA+) cells in healthy controls (blue) and in localized prostate cancer patients (gray). Contradictory, in metastatic prostate cancer samples (red), mean CD45 levels in TRA + cells are lower. Shown are the normalized mean and standard deviation for one example per group. (B) shows the data set for healthy controls (blue), metastatic (red) and localized (gray) prostate cancer samples and for the patient who progressed (orange) analyzing the normalized CD45 intensity differences calculated from TRA + minus TRA-cells. Note that only samples were included which contained at least three TRA + cells. Positive differences indicate overall higher CD45 levels in TRA + cells. (C) Receiver operating characteristics (ROC) curves show diagnostic value and area under the curve comparing the CD45 intensity differences from healthy controls and metastatic prostate cancer patients (black curve) and the differences from localized and metastatic prostate cancer patients (gray curve).

Since we found that the TRA+/CD45^low^ cell population is frequently present in metastatic prostate cancer patients, we next wanted to analyze the overall CD45 levels in TRA negative (TRA-) compared to TRA + cells. For this purpose, we analyzed the mean CD45 fluorescence intensities for all TRA- and all TRA + cells separately for each sample. First, we plotted the normalized mean CD45 intensities for one representative data set from each cohort (Figure 6A). In the healthy control (Figure 6A, blue) as well as in the sample from a patient with localized prostate cancer (Figure 6A, gray) the mean CD45 levels were higher in TRA + cells compared to cells negative for TRA. Interestingly, this was the opposite in the sample from one of the metastatic prostate cancer patients (Figure 6A, red). Here, the TRA + cells overall showed a lower mean CD45 intensities. Based on this, we calculated for all samples at least containing three TRA + cells, the CD45 intensity differences between TRA+ and TRA-cells (Figure 6B). If the TRA + cells had higher CD45 levels the difference showed a positive number. A negative difference indicates that the TRA + cells exhibited lower CD45 levels compared to TRA-cells. Healthy controls (Figure 6B, blue, n = 7) and samples from localized prostate cancer patients (Figure 6B, gray, n = 11) mostly showed a positive value. In contrast, the differences in CD45 levels from patients with metastatic prostate cancer were very low or even negative (Figure 6B, red, n = 26). The patient who progressed to metastatic prostate cancer showed a negative value of about -0.12 (Figure 6B, orange). ROC curve analysis determined a highly significant diagnostic performance to distinguish between healthy controls and metastatic prostate cancer patients with an AUC of 0.94, a CI between 0.84-1 and a *p*-value < 0.001 (Figure 6C, black curve). More interestingly we compared localized and metastatic prostate cancer samples using ROC curve analysis (Figure 6C, gray curve). This analysis showed a good performance with an AUC of 0.84, a CI between 0.70-0.98 and a *p*-value < 0.002. With this test there was only poor, non-significant discriminating power comparing healthy controls and localized prostate cancer patients (AUC: 0.68, CI: 0.41–0.94, *p*-value = 0.2, data not shown).

## 4 Discussion

Most diagnostic, cell-based tests using blood as a source for analysis focus on the isolation of CTCs. The only FDA–approved CTC-based diagnostic test for advanced prostate cancer is the CELLSEARCH® system [17, 18, 19]. As described before, this test relies on the detection of CTCs from the blood of patients based on epithelial markers [20]. Although these cells are detected in greater number in men with metastatic disease, their importance in cancer progression is uncertain. The transition from an epithelial to a more mesenchymal phenotype highly correlates with cancer metastasis [21, 22, 23, 24]. Several studies have discussed the possibility that capture of CTCs based on epithelial features is likely not optimal to identify cells with metastatic potential [25, 26, 27]. Specifically, mesenchymal-like or stem cell-like CTCs have been linked to relapse and metastasis. For us it was a concern that we detected ≥5 CTCs (EpCAM+/CK+/CD45-) in only two out of 10 analyzed blood samples from metastatic prostate cancer patients. With the goal to develop an assay to discriminate metastatic from localized prostate cancer we decided to move to a more unbiased approach and based our analysis on the staining of whole PBMCs without any pre-selection step.

This is the first study to report the finding of cells within the blood of metastatic prostate cancer patients that are positive for the embryonic marker TRA-1-60. These cells showed almost undetectable CD45 levels. Overall, we want to point out that we don't know the origin of this cell population or if these cells are indeed disseminated cancer cells or even a group of haematopoietic progenitor CD34 + stem cells. For these reasons we refrained throughout the manuscript to call these cells CTCs and rather only referred to them as TRA+/CD45^low^ cells. It would be very interesting in future studies to determine the origin and a potential tumorigenicity of this cell population. As these cells (TRA+/CD45^low^) were found frequently in men with metastatic disease and less in patients with localized prostate cancer, this population of cells may be of use as a diagnostic but also as a surveillance tool. At this point we can only speculate about the origin of these cells. To further determine the nature of the cells more detailed follow-up studies would be required.

In our study, we scored one patient metastatic 4 month before the metastatic site was detected by conventional methods. A body scan 2 month before the analyzed blood sample was drawn, appeared also negative for any detectable metastasis. This highlights the potential of TRA-1-60 as a biomarker for metastatic cancer. Overall, the assay described in our study has the potential to be translated into the clinic as a blood-based diagnostic test to identify patients with life-threatening, metastatic prostate cancer who would benefit from harsher treatment options but also to reduce over-treatment and spare patients who show localized disease. One could image that such an assay could be used as a surveillance tool to monitor disease progression especially in patients classified as Gleason 6 where treatment decisions are complex and difficult. Our study provides preliminary basis for the usability of the detection of TRA+/CD45^low^ cells to discriminate between localized and metastatic prostate cancer patients. This study was performed on a limited number of patients and further validations in bigger cohorts need to be performed to evaluate the full potential of the approach. Furthermore, additional experiments would be required to define the nature of this cellular phenotype especially interesting in this regard would be the analysis of this cell population in chemotherapy naïve patient samples. This would help answering the open question of the origin of the cell population. Also, additional staining for prostate specific markers could clarify if these cells disseminated from the primary prostate cancer.

## Declarations

### Author contribution statement

Claudia Schaefer: Conceived and designed the experiments; Performed the experiments; Analyzed and interpreted the data; Wrote the paper.

Yawen Ju, Youngbin Tak: Performed the experiments.

Cesar Vazquez, Edwin Tan: Contributed reagents, materials, analysis tools or data.

Sangyoon J. Han: Analyzed and interpreted the data; Contributed reagents, materials, analysis tools or data.

Jerry Shay, Mats Holmqvist, Gaudenz Danuser, William Schopperle: Conceived and designed the experiments.

Glenn Bubley: Conceived and designed the experiments; Analyzed and interpreted the data; Wrote the paper.

### Funding statement

This work was performed in laboratories constructed with support from NIH grant C06 RR30414 (J.W.S), the Harold Simmons NCI Designated Comprehensive Cancer Center Support Grant (CA142543) (J.W.S) and the Cancer Prevention Institute of Texas (CPRIT) grant RP1225 (C.S., S.H., and G.D.).

### Competing interest statement

YJ, YT, ET, MH and WMS are employed by CureMeta but were not involved in the data collection and analysis. All other authors declare no potential conflicts of interest.

### Additional information

No additional information is available for this paper.
